# Inflammatory Bowel Disease and Neutrophil–Lymphocyte Ratio: A Systematic Scoping Review

**DOI:** 10.3390/jcm10184219

**Published:** 2021-09-17

**Authors:** Blake O. Langley, Sara E. Guedry, Joshua Z. Goldenberg, Douglas A. Hanes, Jennifer A. Beardsley, Jennifer Joan Ryan

**Affiliations:** 1 Helfgott Research Institute, National University of Natural Medicine, Portland, OR 97201, USA; blangley@nunm.edu (B.O.L.); sara.guedry@nunm.edu (S.E.G.); jgoldenberg@nunm.edu (J.Z.G.); dhanes@nunm.edu (D.A.H.); 2 Independent Researcher, Seattle, WA 98115, USA; beardsleyja@gmail.com

**Keywords:** neutrophil–lymphocyte ratio, biomarker, inflammatory bowel disease, Crohn’s disease, ulcerative colitis, disease activity, mucosal healing

## Abstract

Neutrophil–lymphocyte ratio (NLR) is a biomarker of the systemic inflammatory response. The objective of this systematic scoping review was to examine the literature on NLR and inflammatory bowel disease (IBD). PubMed, Embase, Cochrane CENTRAL, CINAHL, ClinicalTrials.gov, Cochrane Specialized Register, DOAJ, PDQT, Biosis Citation Index, Scopus, and Web of Science were systematically searched. A total of 2621 citations yielding 62 primary studies were synthesized under four categories: distinguishing patients with IBD from controls, disease activity differentiation, clinical outcome prediction, and association of NLR with other IBD biomarkers. Thirty-eight studies employed receiver operating characteristic (ROC) curve analysis to generate optimal NLR cutpoints for applications including disease activity differentiation and prediction of response to treatment. Among the most promising findings, NLR may have utility for clinical and endoscopic disease activity differentiation and prediction of loss of response to infliximab (IFX). Overall findings suggest NLR may be a promising IBD biomarker. Assessment of NLR is non-invasive, low cost, and widely accessible given NLR is easily calculated from blood count data routinely and serially monitored in patients with IBD. Further research is justified to elucidate how evaluation of NLR in research and clinical practice would directly impact the quality and cost of care for patients living with IBD.

## 1. Introduction

The prevalence of inflammatory bowel disease (IBD) is increasing worldwide [1]. Estimated costs of the disease have rapidly increased [2,3], and rising expenditures contribute to health disparities [3,4,5,6]. The costs of care, both direct and out-of-pocket, are estimated to be up to three-fold higher in patients with IBD compared to non-IBD controls [3]. The expense of biologic drugs (e.g., infliximab) in particular, which can cost up to roughly $26,270 USD per patient annually (This value is generated from Beilman et al.’s [7] $33,000 CAD estimate using Olsen & Associates (OANDA) currency converter on 27 July 2021. https://www1.oanda.com/currency/converter/) [7], is a key predictor of these rising expenditures [3]. Predictably, high out-of-pocket costs for testing and treatment, for insured and uninsured patients alike [8], can limit access to care, particularly in low and lower-middle income countries [6]. Accurate and cost-effective approaches for IBD management are priorities for patients, clinicians, payers, and other key stakeholders [2].

The two eminent forms of IBD, Crohn’s disease (CD) and ulcerative colitis (UC), are characterized by intermittent or sustained ulceration in the alimentary tract [9] and by chronic, systemic inflammation [10,11]. It is well established that the systemic inflammatory response is associated with peripheral alterations in the two most abundant types of white blood cells (WBC), specifically, relative increases in neutrophils with relative decreases in lymphocytes [10]. In the intestinal tissue of patients with IBD, the transepithelial migration of neutrophils is a hallmark of the disease [10] and closely correlates with disease activity [12,13]. This chronic neutrophilic infiltration compromises the integrity of the epithelial barrier and promotes the formation of cryptitis and crypt abscesses [13].

The gold standard for diagnosis and monitoring of disease activity in patients with IBD is endoscopy [14]. However, routine endoscopy has drawbacks related to invasiveness, feasibility of use for long-term follow up, cost, and inter-observer variability [14,15,16,17]. Of timely relevance, endoscopic procedures were reduced by as much as 95% for patients with IBD during the coronavirus 2019 (COVID-19) pandemic, as endoscopy is a high-risk procedure for SARS-CoV-2 transmission [18].

Specific adjunctive inflammatory biomarkers play key roles at nearly every point in the management of IBD, since they can be used to distinguish patients with IBD from controls, differentiate active from inactive disease, and predict clinical outcomes, such as response to therapy, including mucosal healing [19,20,21]. Biomarkers widely used in IBD care include C-reactive protein (CRP), erythrocyte sedimentation rate (ESR), blood cell counts, fecal calprotectin, and fecal lactoferrin [21,22,23]. However, some of these markers have limitations; for example, despite the high sensitivity of calprotectin and lactoferrin as markers of endoscopically active IBD [22], some patients may prefer blood-based over stool-based testing [19]. This preference could potentially be related to a reluctance to handle fecal material, particularly for serial assessments [24]. Research and development efforts focused on identification of new, noninvasive, cost-effective, adjunctive biomarkers with utility in IBD care are considered a priority [22,23,25]. Endeavors related to proteomic [26], lipidomic [26], microbial [17], and precision [25] IBD biomarker discovery are currently underway.

Neutrophil–lymphocyte ratio (NLR) is a readily available hematologic biomarker of the systemic inflammatory response. Emerging evidence indicates NLR may be a promising biomarker with utility for several conditions including rheumatoid arthritis [27], cardiovascular disease [28,29], cerebrovascular incidents [30], metabolic syndrome [31], various cancers [32,33], and SARS-CoV-2 infection [34]. The NLR value is easily obtained from routine blood count data by dividing the absolute neutrophil count by the absolute lymphocyte count. Data for determining NLR can be readily obtained for nearly any patient under care for IBD given that, per consensus guidelines, complete blood counts are regularly evaluated in patients with IBD to screen for and monitor iron deficiency and anemia [21]. Studies have suggested the potential utility of NLR for the screening, diagnosis, and management of patients with IBD [35,36,37], but current evidence is mixed and no reviews, systematic or otherwise, have been reported on NLR and IBD to date. The aim of this systematic scoping review was to compile all available literature on NLR and human subjects with IBD to describe what has been reported and to inform future research on this topic.

## 2. Materials and Methods

### 2.1. Protocol

The protocol was informed by the processes outlined by Munn (2018) [38] and methodological framework from Arksey and O’Malley (2005) [39]. The initial protocol was developed under the guidance of a research team with experience in IBD research, IBD clinical practice, and systematic reviews. The team included a methodologist, a biostatistician, clinician researchers, and a research librarian. The final protocol was approved by the study team and published a priori with Open Science Framework (DOI 10.17605/OSF.IO/HFVDW). Throughout the conduct of the scoping review, the study team followed the Preferred Reporting Items for Systematic reviews and Meta-Analyses extension for Scoping Reviews (PRISMA-ScR) checklist (Supplementary: Preferred Reporting Items for Systematic reviews and Meta-Analyses extension for Scoping Reviews (PRISMA-ScR) Checklist.) to ensure completeness and accuracy of reporting [40].

### 2.2. Inclusion Criteria

As the fundamental purpose of the review was to identify all available clinical data on NLR and IBD, the review included a broad base of inclusion criteria. All clinical research involving human subjects with IBD, CD, and UC that reported data on NLR, calculated from peripheral blood as absolute neutrophil count divided by absolute lymphocyte count, were included. Since some reports did not discriminate between subsets of IBD, any report involving a diagnosis of “IBD” was included. No limitations were placed on study type, sample size, location, year published, age or sex of participants, or language of publication.

### 2.3. Search Strategy

The search strategy aimed to identify published and unpublished studies, with no restriction on date. Key search terms were used to develop a comprehensive search strategy by an experienced research librarian with input from the study team. The search strategy was then peer reviewed through the Peer Review of Electronic Search Strategies (PRESS) Forum. The final search strategies can be found in Appendix A. Searches were conducted using standard and grey literature databases and executed through PubMed, Embase, Cochrane Central Register of Controlled Trials (Cochrane CENTRAL), Cumulative Index to Nursing and Allied Health Literature (CINAHL), ClinicalTrials.gov, International Standard Randomized Controlled Trial Number (ISCRTN), World Health Organization International Clinical Trials Registry Platform (WHO ICTRP), Cochrane Specialized Register: “Gut”, Directory of Open Access Journals (DOAJ), ProQuest Dissertations and Theses (PQDT), Biosis Citation Index, Scopus, and Web of Science. The original search was performed on 21 July 2020 and updated on 19 June 2021.

### 2.4. Article Review and Data Extraction

Article titles and abstracts were downloaded to EndNote reference manager and reviewed to remove duplicates [41]. Articles were then uploaded to Covidence and independently reviewed by two authors (JR and BL) by title and abstract, followed by full text review to determine relevancy and eligibility [42]. Any disagreements were resolved by consensus or adjudicated by a third author (JG). For studies reported in more than one publication, the publication with the most complete data set was considered the index reference, but data from all associated citations were used. The index reference and any linked references are summarized in Table 1.

Data were extracted to a piloted extraction form using Microsoft Excel (2021) [43], independently and in duplicate (BL and either JR or SG). Any disagreements were resolved by consensus or adjudication (JG). Data were extracted as available for: study location, sample size, and country; participant characteristics (age, sex, diagnosis); aims and methods; and relevant outcome data (such as summary NLR values, cutpoints, and relationship to other biomarkers). For articles that did not report a specific study design, design was determined by a review of the methodology was conducted by two authors (BL and DH) with any disagreement adjudicated by a third author (JG).

It should be noted there are myriad disease activity/severity instruments used in IBD diagnosis and monitoring. These instruments may reflect endoscopic findings, biomarker values, and clinical signs and symptoms. Commonly used examples are the Crohn’s Disease Endoscopic Index of Severity (CDEIS) [44], Mayo Endoscopic Score (MES) [45], Crohn’s Disease Activity Index (CDAI) [46], Harvey–Bradshaw Index (HBI) [46], Truelove and Witts Criteria (TWC) [46,47], Partial Mayo Score (PMS) [46,47], and the Montreal Classification for IBD [48]. Instruments used in each included study are noted throughout the Results section.

Studies were grouped according to diagnosis as: (1) CD, (2) UC, (3) CD and UC, (4) IBD (i.e., not identified as CD or UC). Data were summarized according to the objectives outlined above and reported in narrative format.

## 3. Results

### 3.1. Description of Studies

**Figure 1:**
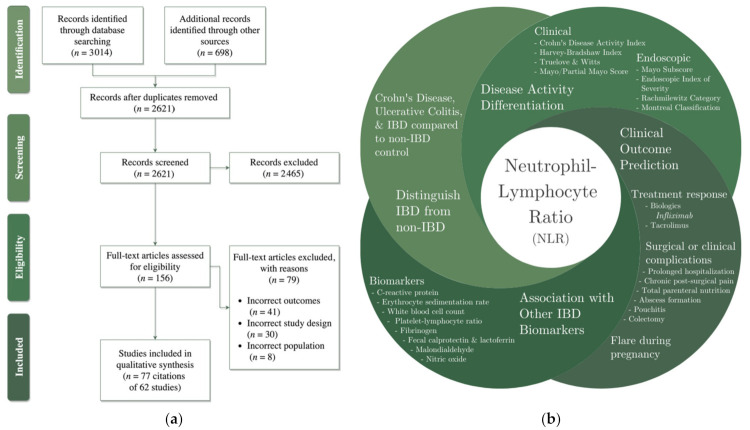
(**a**) PRISMA flow diagram of study selection; (**b**) identified categories of utility for neutrophil–lymphocyte ratio.

Seventy-seven citations met the inclusion criteria and represented 62 primary studies (Figure 1a). The studies varied in size (9 to 4739 participants), originated from 19 countries, and used a variety of methodological designs (Table 1). In 48% of the studies (*n* = 29) the disease assessed was UC, in 27% (*n* = 17) CD was assessed, and 15% (*n* = 9) assessed IBD without identifying a specific subset. Notably, 10% (*n* = 6) assessed both CD and UC, separately, within a single study.

Objectives for assessing NLR in primary studies included generating cutpoint thresholds (*n* = 38), distinguishing patients with IBD from non-IBD controls (*n* = 25), differentiating clinical disease activity (*n* = 23), testing for an association between NLR and IBD biomarkers (*n* = 18), differentiating endoscopic/mucosal activity (*n* = 17), predicting treatment response or loss of response (*n* = 14), and predicting clinical outcomes (*n* = 13). Most studies assessed NLR for more than one purpose (*n* = 44). Four main themes that emerged during article review and data extraction are described in Figure 1b.

Of thirty-eight studies that generated cutpoint values for NLR, the majority (*n* = 31) employed receiver operating characteristic (ROC) curve analysis to identify the optimal cutpoint [49]. All values and analytical statistics for cutpoints are reported in Table 2.

### 3.2. NLR to Distinguish IBD from Non-IBD

Twenty-five studies assessed the utility of NLR to distinguish patients with IBD from non-IBD controls. Twenty-three studies compared NLR values of individuals with an IBD diagnosis to otherwise healthy controls, one study made a comparison to individuals with irritable bowel syndrome (IBS), and one study made a comparison to patients with *C. difficile* infection. Ten of the twenty-five studies also explored potential cutpoints using ROC curve analysis for the same purposes.

#### 3.2.1. NLR Differences in IBD vs. Non-IBD

Twenty-four studies compared NLR values in patients with CD [35,36,50,51,52,53,54,55,56], UC [35,36,56,57,58,59,60,61,62,63,64,65,66,67,68], or IBD to NLR values in non-IBD controls [69,70,71].

CD: Ahmad et al. [36], Chen et al. (2018) [52], Feng et al. [54], Gao et al. (2015) [55], and Zhou et al. [50] reported significantly higher NLR values in patients with CD than in healthy controls (*p* < 0.05, =0.034, <0.01, <0.001, and <0.001, respectively). Zhang et al. (2017) [56] and Acarturk et al. [35] reported significant differences in NLR between patients with active CD compared to healthy controls (*p* < 0.005 and <0.001, respectively), but not between patients with inactive disease compared to controls (both *p* > 0.05). Eraldemir et al. (2016) [53] reported NLR was higher in both active and inactive CD compared to healthy controls (*p* = 0.034 and *p* < 0.001, respectively). Bou Jaoude et al. [51] compared NLR values of patients with CD (77.6% in remission) to patients with IBS and found no difference (*p* = 0.907).

UC: Ahmad et al. [36], Dong et al. [63], and Eraldemir et al. (2014) [64] noted higher NLR values in UC compared to controls (all *p* ≤ 0.05). Jeong et al. (2021) [66], Torun et al. [68], Hanai et al. [65], and Zhang et al. (2021) [57] also noted higher NLR values in UC compared to healthy controls (all *p* < 0.001). Demir et al. [62], Acarturk et al. [35], Celikbilek et al. [60], Michalak et al. [58], and Okba et al. [67] reported NLR was higher in active UC compared to healthy controls (*p* = 0.005, <0.001, <0.001, <0.001, and <0.001, respectively). Zhang et al. (2017) [56] found higher NLR in clinically active UC compared to control (*p* < 0.005) and, Akpinar et al. [59] reported higher NLR in patients with endoscopically active UC compared to control (*p* < 0.001). Cherfane et al. [61] reported higher NLR in active UC compared to patients with *C. difficile* infection (*p* < 0.0001). Ahmad et al. [36] additionally noted higher NLR values in UC compared to CD (*p* ≤ 0.05).

IBD: Chalmers et al. [69] reported higher NLR in pediatric patients with IBD compared to those without IBD (*p* < 0.0001). Guthrie et al. [70] reported a significant difference in colorectal cancer patients with IBD compared to colorectal cancer patients without IBD (*p* < 0.001). However, Ndulue et al. [71] found no significant difference in NLR in adults from the National Health and Nutrition Examination Survey (NHANES) with self-reported CD or UC compared to those without self-reported IBD.

##### NLR Cutpoints to Distinguish IBD from Non-IBD

Proposed NLR cutpoints generated by ROCrve analysis and respective analytical statistics are summarized by disease in Table 2.

CD: NLR was assessed to distinguish patients with CD from healthy controls by Chen et al. (2018) [52] using a cutpoint at 2.85 (69.2% sensitivity, 76.2% specificity); by Feng et al. [54] using a cutpoint at 2.72 (68.3% sensitivity, 75.9% specificity); and by Gao et al. (2015) [55] using a cutpoint at 2.13 (82.7% sensitivity, 76.9% specificity, 80.9% overall accuracy). Uniquely, Bou Jaoude et al. [51] attempted to distinguish CD from IBS using a cutpoint of 1.98, though it did not reach statistical significance (*p* = 0.177).

UC: NLR was assessed to distinguish patients with UC from healthy controls by Cherfane et al. [61] using a cutpoint at 2.60 (70.0% sensitivity, 63.0% specificity) and by Zhang et al. (2021) [57] using a cutpoint at 2.66 (75.0% sensitivity, 82.6% specificity). Additionally, Cherfane et al. [61] determined that an NLR value of 3.10 may distinguish patients with UC from patients with C. difficile infection (70.0% sensitivity, 65.0% specificity).

IBD: Jeong et al. (2018) [72] found that a cutpoint at 1.80 (70.7% sensitivity, 73.3% specificity) could distinguish adult IBD from healthy controls, and Chalmers et al. [69] reported a cutpoint at 2.37 (67.0% sensitivity, 85.0% specificity) for distinguishing pediatric IBD from healthy controls.

### 3.3. NLR to Differentiate Disease Activity in IBD

Thirty-four studies explored the relationship between NLR and disease activity by assessing statistical differences in group NLR means, either by employing ROC curve analysis to generate optimal NLR cutpoints to differentiate disease activity or by using regression analysis to ascertain the strength of relationships between NLR values and activity scores. Most studies assessed clinical activity (*n* = 24), followed by endoscopic activity (*n* = 17), or both within the same study (*n* = 6).

#### 3.3.1. Relationship between NLR and Clinical Disease Activity

CD: Eight studies assessed potential differences in NLR between patients with clinically active or inactive CD using the following scores:

Crohn’s Disease Activity Index (CDAI): Eraldemir et al. (2016) [53], Zhang et al. (2017) [56], Ben Jeddi et al. [73], and Chen et al. (2020) [37] reported higher NLR in active disease (*p* = 0.034, <0.05, =0.004, and <0.001, respectively), whereas Gao et al. (2015) [55] did not find a difference in NLR between active and inactive disease (*p* > 0.05). Chen et al. (2020) [37] additionally found a positive correlation of NLR with CDAI (r_s_ = 0.451, *p* < 0.001).

Harvey–Bradshaw Index (HBI): Acarturk et al. [35], Gold et al. [74], and Xu et al. [75] found NLR was higher in active CD compared to inactive CD (*p* < 0.001, <0.001, and 0.001, respectively).

UC: Nineteen studies assessed potential differences in NLR between patients with clinically active UC or inactive UC using the following scores:

Truelove and Witts Criteria (TWC) or modified TWC: Posul et al. [76], Demir et al. [62], Fidan et al. [77], Acarturk et al. [35], Celikbilek et al. [60], Torun et al. [68], and Yamamoto-Furosho et al. [78] found NLR was higher in active UC compared to inactive UC (*p* < 0.05, =0.005, <0.002, <0.001, <0.001, <0.001, and <0.001 respectively). Xu et al. [75] noted no differences in NLR between active and inactive UC (*p* = 0.273).

Mayo Score and Partial Mayo Score (PMS): Chen et al. (2020) [37], Cherfane et al. [61], Gold et al. [74], and Okba et al. [67] reported higher NLR in active disease as defined by Mayo score (all *p* < 0.001); and Ovidiu et al. [79] noted similar findings (*p* < 0.01). Dong et al. [63] and Jeong et al. (2021) [66] reported higher NLR in moderate-to-severe UC compared to mild UC (*p* < 0.05 and <0.001, respectively) by Mayo score. Chen et al. (2020) [37] additionally correlated NLR with Mayo score (r_s_ = 0.393, *p* < 0.001) whereas Nishida et al. (2017) [80] found no relationship between NLR and PMS (*p* > 0.05).

Colitis Activity Index (CAI): Abedi Manesh et al. [81] found a positive correlation between NLR and CAI (*p* < 0.05).

Simple Clinical Colitis Activity Index (SCCAI): Zhang et al. (2017) [56] did not find a significant difference in NLR between patients with active or quiescent UC (*p* > 0.05).

Using a newly developed “noninvasive activity score”, Hanafy et al. [82] reported NLR was higher in newly diagnosed patients with UC or in relapse, compared to patients in remission.

##### NLR Cutpoints to Differentiate Clinical Disease Activity

Proposed NLR cutpoints generated by ROC curve analysis and respective analytical statistics are summarized by disease in Table 2.

CD: NLR was assessed to potentially distinguish patients with active CD from patients with inactive CD in the following studies:

Ben Jeddi et al. [73]—cutpoint at 1.57
Zhang et al. (2017) [56]—cutpoint at 1.95 (95.5% sensitivity, 56.1% specificity)
Eraldemir et al. (2016) [53]—cutpoint at 2.58 (69.6% sensitivity, 76.0% specificity)
Acarturk et al. [35]—cutpoint at 3.2 (81.0% sensitivity, 59.0% specificity, *p* < 0.001)
Chen et al. (2020) [37]—cutpoint at 3.32 (65.9% sensitivity, 75.9% specificity)
Zhang et al. (2017) [56]—cutpoint at 5.35 to discriminate between mild-to-moderate and severe disease (75.0% sensitivity, 92.9% specificity, *p* = 0.02)
Xu et al. [75] found no significance in any NLR cutpoint value to discriminate between active and inactive CD (AUC = 0.631)

UC: NLR was assessed to potentially differentiate patients with active UC from patients with inactive UC in the following studies:

Hanafy et al. [82]—cutpoint at 2.35 (74.0% sensitivity, 86.0% specificity)
Demir et al. [62]—cutpoint at 2.39 (48.6% sensitivity, 77.5% specificity)
Chen et al. (2020) [37]—cutpoint at 2.40 (76.2% sensitivity, 84.5% specificity)
Celikbilek et al. [60]—cutpoint at 2.47 (53.9% sensitivity, 63.2% specificity)
Acarturk et al. [35]—cutpoint at 3.1 (78.0% sensitivity, 69.0% specificity, *p* < 0.001)
Zhang et al. (2017) [56]—cutpoint at 3.29 (47.4% sensitivity, 93.9% specificity)
Dong et al. [63]—cutpoint at 4.70 (61.0% sensitivity, 86.0% specificity)

Cherfane et al. [61] reported no NLR cutpoint stratified inactive from active UC, nor from mild-to-moderate to severe UC (both *p* > 0.05). Zhang et al. (2017) [56] also did not identify a cutpoint to differentiate mild-to-moderate from severe UC (*p* = 0.517).

Xu et al. [75] found no cutpoint for NLR to distinguish active from inactive UC (AUC = 0.625).

#### 3.3.2. Relationship between NLR and Endoscopic Disease Activity

CD: Five studies assessed for potential relationships between NLR and endoscopic activity using the following scores:

Simple Endoscopic Score for Crohn’s Disease (SES-CD): Zhou et al. [50] reported that NLR correlated with mucosal healing (SES-CD ≤ 2; r_s_ = −0.31, *p* < 0.001). However, using multivariate regression analysis, Crispino et al. [83] reported that no association was found between NLR and SES-CD prior to starting biologics (*p* = 0.859).

Crohn’s Disease Endoscopic Index of Severity (CDEIS): Bou Jaoude et al. [51] did not find a relationship between NLR and endoscopic disease activity (OR = 1.128, 95% CI 0.680–1.871, *p* = 0.642). Kang et al. [84] did not find a relationship between NLR and lesion location (ileal, colonic, ileocolonic, and/or upper gastrointestinal; *p* > 0.05).

Nassri et al. [85] did not note a difference in NLR when stratifying by histological disease activity (*p* = 0.4; sample size unspecified).

UC: Eleven studies assessed for potential relationships between NLR and endoscopic activity using the following scores:

Mayo Endoscopic Score (MES) and Montreal classification: Dorobăţ et al. [86] reported that higher MES was associated with higher NLR (5.2 in active disease vs. 1.9 in remission) and that NLR was an independent predictor of endoscopic disease activity (68% sensitivity, 71% specificity). Hanafy et al. [82] found a relationship between NLR and MES (r_s_ = 0.68, *p* < 0.001) but not disease extent or lesion location (r_s_ = 0.19, *p* = 0.2). Zhang et al. (2021) [57] did not find a correlation between NLR and MES (r_s_ = 0.068, *p* = 0.375) though the correlation with disease extent approached significance (r_s_ = 0.146, *p* = 0.056). Okba et al. [67] reported NLR was a predictor of active vs. inactive endoscopic disease (OR 35.23, 95% CI 7.54–165.244, *p* < 0.001) and was higher in patients with pancolitis compared to those with less extensive disease (*p* < 0.001). Yamamoto-Furosho et al. [78] reported a correlation between NLR and endoscopic findings (r_s_ = 0.310, *p* < 0.001), specifically according to MES (r_s_ = 0.439, *p* < 0.001) and Montreal classification (r_s_ = 0.208, *p* < 0.001). Bertani et al. (2020) [87] associated the presence of active ulcers (classified by MES) at baseline (prior to anti-TNF treatment) with higher NLR compared to those without active ulcers (*p* = 0.002) and reported a baseline NLR cutpoint at 2.06 could predict mucosal healing (MES ≤ 1) following 54 weeks of treatment with biologics (60% sensitivity, 79% specificity). Bertani et al. (2019) [88] used a 2-point or greater reduction in the MES to define mucosal healing and observed a correlation between mucosal healing and NLR at baseline (*p* < 0.05) and after eight weeks of anti-TNF treatment (*p* < 0.05) with an NLR cutpoint of 2.33 to predict mucosal healing (80% sensitivity and 67% specificity).

Degree of Ulcerative Colitis Endoscopic Index of Severity (DUBLIN): Zhang et al. (2021) [57] did not find a correlation between NLR and DUBLIN, though the findings approached significance (r_s_ = 0.139, *p* = 0.068); furthermore, they determined an NLR value of 2.67–4.23 was a risk factor independently associated with DUBLIN > 3 (OR = 2.96, *p* = 0.047).

Rachmilewitz Endoscopic Activity Index (EAI): Akpinar et al. [59] reported NLR was higher in endoscopically active UC compared to remission and control (both *p* < 0.001) and positively correlated with the Rachmilewitz EAI score (r_s_ = 0.321, *p* = 0.001).

UC Endoscopic Index of Severity (UCEIS): Dong et al. [63] reported a significant correlation between NLR and UCEIS (*p* < 0.05). Zhang et al. (2021) [57] did not find a significant correlation between NLR and UCEIS (r_s_ = 0.130, *p* = 0.088).

Other: Jardak et al. [89] reported significantly higher NLR in endoscopically active compared to inactive UC (*p* = 0.042), yet Cherfane et al. [61] reported no significant difference in NLR when comparing endoscopically active and quiescent UC (*p* = 0.144); however, neither report specified the tool used for differentiating disease activity.

IBD: Jeong et al. (2018) [72], assessed for a potential relationship between NLR and endoscopic activity in unspecified IBD and found no significant correlation.

##### NLR Cutpoints to Differentiate Endoscopic Disease Activity

CD: Zhou et al. [50] determined an NLR cutpoint at 4.45 could predict mucosal healing (defined as SES-CD 0-2; 83.9% sensitivity, 46.9% specificity).

UC: Yamamoto-Furosho et al. [78] reported an NLR value > 2.09 predicted endoscopic activity according to Mayo score (63.9% sensitivity, 58.8% specificity), and Akpinar et al. [59] determined an NLR cutpoint at 2.42 distinguished endoscopic activity from remission states using Rachmilewitz EAI (76.0% sensitivity, 70.2% specificity, *p* = 0.003).

### 3.4. NLR to Predict Clinical Outcomes

The potential of NLR to inform clinicians of the possibility of improved or diminished response to treatment, extended post-operative hospital stay, and development of complications from disease processes was explored in twenty studies.

#### 3.4.1. NLR and IBD Treatment

##### Shift in NLR and Prediction of Response to Biologics (Including Anti-TNF)

CD: Wlodarczyk et al. [90] reported that a baseline NLR value ≤ 4.07 predicted sustained response to IFX with 80% sensitivity and 87% specificity, while an NLR value of ≥3.67 at week 14 predicted loss of response to IFX with 67% sensitivity and 80% specificity. Ben Mustapha et al. [91] found higher baseline (>4.00) and week 14 (>3.50) NLR values predicted loss of response to IFX (80% and 72% sensitivity and 80% and 70% specificity, respectively). Gao et al. (2020) [92] noted that, after successful IFX induction therapy, NLR values at week 14 (>2.75) had 93.3% sensitivity and 84.6% specificity as an independent predictor of loss of response to therapy (HR 1.851, 95% CI 1.096–3.026, *p* = 0.021) with a significantly reduced relapse-free survival rate. Crispino et al. [83] found that compared to non-responders, patients who had achieved endoscopic remission after 52 weeks of treatment with biologics had lower mean NLR values at baseline (*p* = 0.02); an NLR cutpoint of 1.55 predicted endoscopic remission (AUC 0.64, *p* = 0.003).

UC: Michalak et al. [58] found that NLR decreased significantly after three doses of infliximab (IFX) induction therapy (*p* < 0.001). Nishida et al. (2017) [80] reported higher baseline NLR (≥4.49) as an independent prognostic factor for loss of response to IFX therapy with 78.6% sensitivity and 78.3% specificity (HR 3.86, 95% CI 1.20–12.4, *p* = 0.023) while no trend was reported after therapy initiation. In primary non-responders or corticosteroid users, Bertani et al. (2019) [88] found higher baseline NLR (≥2.33) predicted clinical remission of UC during anti-TNF (IFX, adalimumab, or golimumab) therapy according to PMS reduction ≥ 2 (90% sensitivity, 65% specificity) and mucosal healing according to MES reduction ≥ 1 (80% sensitivity, 67% specificity). In a subsequent report, Bertani et al. (2020) [87] found NLR was lower in patients who achieved clinical remission, both at baseline and eight weeks, with the same anti-TNF treatments (*p* = 0.0005 and 0.0001, respectively). In patients treated with IFX salvage for steroid-refractory acute severe UC, Andrew et al. [93] reported a near-significant correlation between baseline NLR and requirement for maintenance IFX (*p* = 0.06).

IBD: Stefanidis et al. [94] suggested the potential of NLR as a predictor of treatment response after they determined NLR decreased significantly after IFX treatment two- and six-weeks post-infusion (*p* < 0.001); the decrease did not remain significant at 22 weeks (*p* < 0.01 considered significant).

##### Glucocorticoids and Steroids

CD and UC: The team of Gold and Gordon et al. [74,95] noted NLR values were higher in patients with IBD taking glucocorticoids (*p* < 0.01) and in patients with active compared to inactive CD taking steroids (*p* < 0.01), but not in patients with active compared to inactive UC taking steroids (*p* = 0.12).

##### Other Treatments

UC: Nishida et al. (2019) [96] reported lower baseline NLR (≤5.84) as an independent predictor of clinical relapse with tacrolimus therapy in UC with 62.5% sensitivity and 66.7% specificity (HR 0.82, 95% CI 0.72–0.94, *p* < 0.01), complicated by the potential of concomitant therapy influence on NLR levels. Hanai et al. [65] reported lower NLR in a small sample of patients with active UC receiving Adacolumn adsorptive granulocyte and monocyte apheresis (GMA) therapy (*p* = 0.06) though no predictive cutpoint was assessed. Abedi Manesh et al. [81] reported that in patients with UC, vitamin A injections over a 45-day study period resulted in a “not considerable” decrease in NLR.

IBD: In a pilot study, Ryan et al. [97] reported a near-significant decrease in NLR after 12 weeks of daily administration of a botanical-containing nutrition support formula in patients with CD or UC (*p* = 0.061).

#### 3.4.2. NLR to Predict Length of Post-Operative Hospital Stay and IBD Complications

Eleven studies employed NLR as a potential tool to predict post-surgical hospital stay or development of various complications in CD [84,98,99,100,101], UC [93,102,103,104,105], or IBD [106,107].

CD: Two studies by Gur et al. (2018 and 2020) [100,101] found higher NLR values were significant predictors of postoperative length of hospital stay (*p* < 0.05) and of patients requiring surgical intervention in lieu of regular medical treatment (*p* = 0.007). In patients who presented to the emergency department, Khoury et al. [98] determined NLR to be a significant predictor of abscess formation. Kang et al. [84] generated a cutpoint of 4.1 and found NLR predicted risk of postoperative complications with 70% sensitivity and 56.4% specificity (OR 2.782, 95% CI 1.042–7.425, *p* = 0.041), primarily respiratory infection (*p* = 0.046). However, in patients who underwent intestinal resection for symptomatic CD, Argeny et al. [99] found preoperative NLR values were lower in patients who experienced post-surgical complications (r_s_ = 0.1041, *p* = 0.0446). Conversely, Argeny et al. [99] also determined that NLR values were higher in patients with acute indication for surgery (*p* = 0.037), in those presenting with abscesses (*p* = 0.025), those with inflammatory masses (*p* = 0.029), and those with malignancy in resected specimens (*p* = 0.023). On the other hand, Argeny et al. [99] did not note a difference in NLR values between those with stenosis (*p* = 0.134) or fistula (*p* = 0.153).

UC: Abotaga et al. [102] reported that higher NLR values were correlated with length of hospital stay (r^2^ = 0.1703, *p* < 0.001) with a stronger correlation in white (r^2^ = 0.2991, *p* = 0.003) compared to black patients (r^2^ = 0.1112, *p* = 0.502); a near-significant relationship between NLR and an increased number of UC flares was also reported (*p* = 0.07). Nishida et al. (2020) [104] reported that in patients who underwent ileal pouch-anal anastomosis (IPAA), both a continuous NLR value (*p* = 0.03) and a cutpoint of 2.15 (72.2% sensitivity, 67.7% specificity) were associated with the development of pouchitis. NLR was additionally identified as a prognostic factor for the development of pouchitis using inverse probability of treatment weighting (HR 3.60, 95% CI 1.31–9.89, *p* = 0.01) [104]. In a subset of patients with UC who underwent IPAA, Fleshner et al. [105] determined that higher NLR was associated with increased rates of de novo CD as a defined post-surgical complication (*p* = 0.03). In patients treated with IFX salvage for steroid-refractory acute severe UC, Con et al. [103] reported a weak but significant correlation between day 3 NLR and colectomy within twelve months (*p* = 0.020); NLR value upon admission and on day 1 were not associated with subsequent colectomy (*p* = 0.340 and 0.792, respectively). Similarly, Andrew et al. [93] did not find a correlation between NLR and colectomy after hospital admission prior to or one day following IFX salvage therapy (r = −0.01 and −0.01, respectively; correlation method not specified).

IBD: Parisi et al. [106] found higher preoperative NLR values correlated with chronic post-surgical pain, in patients both with and without IBD (r^2^ = 0.229, *p* = 0.023). In a population with IBD undergoing surgery, Yarur et al. [107] reported preoperative NLR values > 5.0 were predictive of patients requiring total parenteral nutrition (*p* = 0.01). Preoperative NLR values > 5.0 were also shown to be predictive of a longer length of stay for a subset of patients who had been admitted to the intensive care unit (*p* = 0.05); however, NLR was not associated with the actual rate of admission to the intensive care unit [107].

#### 3.4.3. NLR to Predict Flare during Pregnancy

In pregnant women with UC in remission, El-Sadek et al. [108] found that NLR was an independent predictor of flare (*p* = 0.002), that higher early first trimester NLR values were associated with flare during pregnancy (*p* < 0.001), and that an NLR cutpoint of >2.85 was predictive of flare (AUC 0.915, *p* < 0.001).

### 3.5. NLR and Other Biomarkers

Eighteen studies explored the relationship between NLR and other biomarkers for evaluating systemic inflammation in CD [35,53,54,55], UC [35,58,62,63,64,67,68,77,78,82,87,88,108], and IBD [72,109]. Biomarkers assessed included those widely used in IBD care such as CRP, ESR, total WBC count, fecal calprotectin, and fecal lactoferrin. More investigational IBD biomarkers, such as platelet-lymphocyte ratio (PLR), fibrinogen, and malondialdehyde reflect oxidative stress levels, while nitric oxide levels assess epithelial involvement (Table 3).

CD: Four studies reported on potential relationships between NLR and other blood-based biomarkers in patients with CD. Feng et al. [54] found significant correlations between NLR and ESR (r_s_ = 0.43, *p* < 0.01) and NLR and CRP (r_s_ = 0.39, *p* < 0.01). Gao et al. (2015) [55] reported NLR correlated with CRP (r_s_ = 0.327, *p* < 0.001) and total WBC count (r_s_ = 0.493, *p* < 0.001), but not with ESR. In patients with active CD, Acarturk et al. [35] reported a significant correlation between NLR and total WBC count (r_s_ = 0.242, *p* ≤ 0.001); however, this relationship was not observed in inactive CD, and no correlation was observed between NLR and ESR or CRP in active or inactive CD. In patients with active CD, Eraldemir et al. (2016) [53] reported a significant correlation between NLR and malondialdehyde (β = 0.422, 95% CI 0.048–0.796, *p* = 0.029), but no correlation between NLR and ESR or CRP.

UC, Blood-Based Biomarkers: Ten studies reported on potential relationships between NLR and other blood-based biomarkers in patients with UC. Dong et al. [63] reported positive correlations of NLR and ESR and CRP (both *p* < 0.05). Demir et al. [62] reported significant correlations between NLR and ESR (r_s_ = 0.170, *p* = 0.043) and total WBC count (r_s_ = 0.282, *p* = 0.001) as well as with total WBC count in active disease, specifically (r_s_ = 0.360, *p* = 0.002); however, no correlations were found between NLR and CRP in any group, nor with ESR or total WBC count in patients with inactive UC. Okba et al. [67] found correlations between NLR and ESR (r_s_ = 0.556, *p* < 0.001), CRP (r_s_ = 0.789, *p* < 0.001), and total WBC count (r_s_ = 0.324, *p* = 0.012), as well as positive correlations between NLR and ESR (r_s_ = 0.597, *p* = 0.005) and CRP (r_s_ = 0.490, *p* = 0.028) specifically in active UC, but no correlations in inactive UC. Torun et al. [68] reported NLR correlated strongly with ESR (r_s_ = 0.217, *p* = 0.002) and total WBC count (r_s_ = 0.416, *p* < 0.001) but not with CRP or fibrinogen. Fidan et al. [77] reported a positive correlation between NLR and total WBC count (r_s_ = 0.370, *p* < 0.05) and PLR (r_s_ = 0.944, *p* < 0.05) in patients with active UC. Bertani et al. (2019) [88] reported “no correlation was found” between NLR and PLR in patients receiving anti-TNF. In contrast with some of the above findings, Acarturk et al. [35] found no correlations between NLR and CRP, ESR, or WBC in active or inactive UC. Finally, Eraldemir et al. [64] (2014) found a strong correlation between NLR and nitric oxide (r^2^ = 0.593, *p* < 0.001). Michalak et al. [58] reported that NLR and PLR correlated positively (*p* < 0.001), specifically after finishing IFX induction therapy. El-Sadek et al. [108] found that NLR and CRP (r_s_ = 0.418, *p* = 0.030), as well as ESR (r_s_ = 0.522, *p* = 0.005), were correlated, specifically in pregnant women with UC.

UC, Stool-Based Biomarkers: Four studies reported on potential relationships between NLR and stool-based biomarkers in patients with UC. Hanafy et al. [82] found an association between NLR and fecal lactoferrin (*p* < 0.001) and Yamamoto-Furosho et al. [78] reported a highly significant correlation between NLR and fecal calprotectin (r_s_ = 0.347, *p* < 0.001); however, Bertani et al. [88] (2019) reported “no correlation was found” between NLR and fecal calprotectin in patients receiving anti-TNF. In a subsequent report, Bertani et al. [87] (2020) still found no correlation between NLR and fecal calprotectin over eight weeks on anti-TNF (r_s_ = 0.11 at baseline and 0.21 at week 8); however, upon subgroup analysis, NLR values were significantly higher at week 8 in patients with fecal calprotectin > 250 mg/kg compared to patients with fecal calprotectin < 250 mg/kg (*p* = 0.01).

IBD: Two studies reported on the potential relationship between NLR and blood or stool-based biomarkers in patients with IBD, without distinguishing between CD and UC. Jeong et al. [72] (2018) found a positive correlation between NLR and CRP (r^2^ = 0.348, *p* = 0.008) and Messner et al. [109] reported a correlation between NLR and fecal calprotectin (r^2^ = 0.210, *p* ≤ 0.05) with weak to no correlation with CRP, ESR, total WBC count, or platelet count.

## 4. Discussion

This systematic scoping review examined the current evidence on NLR in human subjects with IBD, specifically Crohn’s disease and ulcerative colitis. Major themes identified during the conduct of this review were related to the potential utility of NLR for (1) distinguishing patients with IBD from controls, (2) differentiating between active and inactive disease, (3) predicting clinical outcomes including treatment response and surgical complications, as well as (4) its association with other established or emerging IBD biomarkers.

Although normal ranges for NLR have not yet been clearly defined, it has been reported that average NLR values in healthy individuals are approximately 1.65–1.70 [110,111]. Our review identified numerous studies that reported higher NLR values in patients with IBD versus healthy controls (e.g., Gao et al. (2015) [55] and Zhang et al. (2021) [57]). ROC curve analyses have indicated that NLR cutpoints ranging from 2.13 to 2.85 could distinguish patients with CD from healthy controls [52,54,92], and cutpoints ranging from 2.26 to 4.70 could distinguish patients with UC from healthy controls [63,66].

Most studies that examined the relationship between NLR and *clinical* disease activity found that higher NLR values were associated with higher disease activity, in patients with both CD and UC, with generated cutpoints ranging from 1.57–5.35 and 2.35–4.70, respectively (see Section 3.3.1). Data on NLR and *endoscopic* disease activity in patients with CD were limited to five studies, with four reporting that NLR was *not* associated with endoscopic activity [72,84,85]. However, most studies that reported on NLR and endoscopic disease activity in patients with UC found significant relationships between NLR and endoscopic disease [37,59,63,67,78,82,89] while others explored relationships to active ulcers [87] or mucosal healing associated with treatment [88].

Increased levels of specific biomarkers, such as CRP [19,22,112] and fecal calprotectin [113], are associated with increased disease activity in patients with IBD. Likewise, our review found consistent evidence that NLR could differentiate between active and inactive IBD (e.g., Acarturk et al. [35], Fidan et al. [77], and Hanafy et al. [82]). The identified studies reported cutpoints ranging from 1.58–3.32 and 2.35–4.70 could distinguish patients with clinically active versus inactive CD [35,37,53,56,73] and UC [35,37,56,60,62,63,82], respectively. The broad ranges in these reported cutpoints are potentially related to heterogeneity in the tools used to differentiate clinical disease activity, though this was not assessed within this review.

Several studies evaluated whether NLR was a significant predictor of post-operative length of hospital stay or post-surgical complications, with mixed results. Individual studies suggested NLR may be a predictor of chronic post-surgical pain [106], development of pouchitis following IPAA [104], the requirement for post-surgical total parenteral nutrition [107], and abscess formation in CD patients presenting to the emergency department [98]. One study demonstrated that NLR values were an independent predictor of UC flare during pregnancy [108]. Additional research on NLR in these populations could establish whether NLR has utility for identifying which of these patients may require more careful follow-up.

CRP, ESR, blood cell counts, and the neutrophil-derived stool proteins lactoferrin and calprotectin [114] are among the most widely used biomarkers in IBD care [21,22]. Within both CD and UC studies included in this review, the most common comparisons between NLR and other biomarkers were with CRP, ESR, and total WBC count. Results on potential correlations between NLR and these three markers were not consistent in magnitude and significance. Some studies of NLR and calprotectin or lactoferrin demonstrated strongly significant relationships in patients with UC [78,82,109]. Although some data on potential correlations between NLR and other biomarkers are promising, additional research is necessary to further explore the validity and dependability of NLR as an additional measure to these more established surrogate IBD biomarkers.

As recently reviewed by Privitera et al. [115], valid tools that can be used as predictors and early markers of response to biologic therapies in IBD are currently lacking. Intriguingly, some studies included in this review demonstrated that NLR decreased over time in association with infliximab (IFX) treatment [90,94]. Furthermore, several studies demonstrated that NLR values could be used to predict loss of response to IFX in patients with both CD [90,91,92] and UC [80]. The incidence of primary IFX non-response in this population is reported to be as high as 30% [116] and, as estimated in the ACCENT1 trial, secondary loss of response is reportedly as high as 40% in patients with CD [117]. With heavy costs associated with biologic therapies [7], the integration of NLR into clinical decision-making related to IBD treatment selection could be particularly valuable.

As an illustrative example, using the 30% estimate for primary IFX non-response and a positive likelihood ratio of 6, as calculated from the sensitivity and specificity reported by Gao et al. (2020) [92], IBD patients with NLR values above the generated 2.75 cutpoint would have a 72% chance of loss of response to IFX. Such a potential striking shift in the likelihood of response could reasonably impact clinical decision making. Given the need for tools clinicians can use to identify patients at high risk for loss of response to treatment prior to drug administration, and the current lack of valid predictors and early markers of response to biologic therapies in IBD care [115,118], the collective findings suggest NLR may serve as a useful addition to such algorithms.

This scoping review is the first to assess the literature gap related to summarizing clinical data on NLR and IBD. To our knowledge, this is the first review of any type on NLR and IBD. However, our review has limitations similar to the limitations reported in most scoping reviews [119]. Specifically, despite conducting a thorough search of four databases and ten grey literature sources without language or date restriction, there is a possibility not all relevant studies were identified. Furthermore, a formal assessment of the quality of included studies (i.e., risk of bias assessment) and a quantitative evidence synthesis were not conducted [39,119]. A more targeted evidence quality assessment and potentially quantitative evidence synthesis (i.e., meta-analysis) was beyond the scope of this work and should be addressed in future research on NLR and IBD. This review had many strengths, including registration of the study protocol *a priori*, a study design guided by published scoping review guidelines [39], and reporting of results in line with all essential reporting items within the PRISMA-ScR checklist [40], which together contribute to the strength of this comprehensive review on a topic of rising interest and research priority.

The findings of this review indicate that further research on NLR is justified to better understand whether routine observation of NLR in research and clinical practice, whether solitary or in combination with other markers, could beneficially impact the care of patients with IBD. Like other biomarkers of inflammation (including CRP, ESR, fecal calprotectin, and fecal lactoferrin), confounding factors could potentially impact NLR values in patients with IBD [120]. NLR may be impacted by age [121,122,123], sex [121,123], sex hormone levels [121], menopausal status [121], race and ethnicity [121,123], body mass index [122], blood pressure [122], and smoking status [123,124]. As with other biomarkers in development, the potential impact of confounding factors on the sensitivity and specificity of NLR to identify, measure, or predict IBD clinical outcomes must be considered in subsequent research. Additional challenges to the development and implementation of new IBD biomarkers include needs for cost-effectiveness research and evaluation in prospective studies that assess clinical predictors [19]. However clinical measures used for calculating NLR (i.e., absolute neutrophil and lymphocyte counts) are economical and widely accessible due to being ubiquitously assessed in IBD research and clinical practice and assessment of NLR is relatively noninvasive compared to other common procedures used in the research and management of patients with IBD. Thus, the potential use and development of NLR as a new IBD biomarker may have fewer challenges than other emerging IBD biomarkers in early phases of development [17,25,26].

Based on the promising findings of this review, the authors suggest the following five specific foci of research inquiry into NLR and IBD:

(1) Given that data for calculating NLR are currently readily available in the health records of patients with IBD, more large-scale retrospective studies that leverage existing electronic medical records should be performed to add to the existing literature.

(2) Conversely, most existing studies on NLR in patients with IBD were retrospective with relatively small sample sizes. To reduce potential sources of bias and confounding, more large-scale prospective studies that further explore the utility of NLR as an IBD biomarker, while also assessing clinical predictors, should be performed.

(3) Our review found multiple studies with reasonable homogeneity on several domains, such as the use of NLR to distinguish disease presence, the potential correlation of NLR with other inflammatory biomarkers, and the potential use of NLR to predict therapeutic response. However, to date, no meta-analyses on NLR in patients with IBD have been published; therefore, data from existing studies should be meta-analyzed to improve the precision and quality of effect estimates.

(4) We identified only one study on NLR in pediatric patients [69] and one study on NLR in patients who were pregnant [108]. More research on NLR in pediatric and pregnant populations with IBD are needed as available literature in these vulnerable demographics are promising, yet currently extremely limited.

(5) This review did not identify any existing cost-effectiveness research on the use NLR as a biomarker for IBD. Given the high economic burden of IBD and the relatively low cost to observe NLR, research that examines the economic utility of NLR is warranted.

## 5. Conclusions

The findings of this systematic scoping review highlight the potential utility of NLR as an adjunctive IBD biomarker with broad applications, including differentiation from non-IBD controls, clinical and endoscopic disease activity differentiation, prediction of loss of response to treatment, and prediction of risk of complications. NLR has promise for guiding therapeutic decision making, specifically for predicting loss of response to IFX. In conclusion, NLR is an emerging IBD biomarker with potential utility at nearly every point in IBD management. As a potential IBD biomarker, NLR is particularly advantageous given that it is minimally invasive, economical, and accessible as it is easily calculated from blood count data routinely and serially monitored in patients with IBD. Additional research is justified to better understand if routine observation of NLR in research and clinical practice could beneficially impact the care of patients with IBD.

## Figures and Tables

**Table 1:** Characteristics of included articles.

First Author, Year of Publication	Country	Study Type	Study Sample (*n*)	Condition	Use of Control	Study Aims Pertaining to NLR						
						Differentiate Diagnoses	Differentiate Clinical Activity	Differentiate Endoscopic Activity	Predict Treatment Response	Predict Other Clinical Outcomes	Generate Optimal Cutpoint	Association with Other Biomarkers
*AbediManesh, 2011*	Iran	Uncontrolled, non-randomized trial	43	UC	No		X		X			
*Abotaga, 2009*	USA	Retrospective cohort	62	UC	No					X		
*Acarturk, 2015*	Turkey	Retrospective case-control	83	UC, CD	Yes	X	X				X	X
*Ahmad, 2015*	Iraq	Prospective case-control	90	UC, CD	Yes	X						
*Akpinar, 2018*	Turkey	Retrospective cross-sectional	313	UC	Yes	X		X			X	
*Andrew, 2020*	Australia	Retrospective cohort	72	UC	No				X	X		
*Argeny, 2018*	Austria	Retrospective cohort	373	CD	No					X		
*Ben Jeddi, 2019*	Tunisia	Retrospective cohort	74	CD	No		X				X	
*Ben Mustapha, 2015*	Tunisia	Prospective case-control	47	CD	Yes				X		X	
*Bertani, 2019*	Italy	Retrospective cohort	46	UC	No			X	X		X	X
*Bertani, 2020*	Italy	Prospective cohort	88	UC	No		X	X	X		X	X
*BouJaoude, 2018*	France/ Lebanon	Prospective case-control	116	CD	Yes	X		X			X	
*Celikbilek, 2013*	Turkey	Prospective case-control	54	UC	No	X	X				X	
*Chalmers, 2017*	Scotland	Prospective case-control	182	IBD	Yes	X					X	
*Chen, 2018*	China	Retrospective case-control	120	CD	Yes	X					X	
*Chen, 2020*	China	Retrospective cohort	876	UC, CD	Yes		X				X	
*Cherfane, 2013*	USA	Retrospective cohort	185	UC	Yes	X	X	X			X	
*Con, 2021*	Australia	Retrospective cohort	94	UC	No				X	X		
*Crispino, 2021*	Italy	Retrospective cohort	107	CD	No			X	X		X	
*Demir, 2015*	Turkey	Retrospective cohort	211	UC	Yes	X	X				X	X
*Dong, 2019*	China	Prospective case-control	104	UC	No	X	X	X			X	X
*Dorobăţ, 2018*	Romania	Retrospective cohort	63	UC	No			X				
*El-Sadek, 2019*	Egypt	Retrospective cohort	27	UC	No					X	X	X
*Eraldemir, 2014*	Turkey	Prospective case-control	65	UC	Yes	X						X
*Eraldemir, 2016*	Turkey	Prospective case-control	87	CD	Yes	X	X				X	X
*Feng, 2017*	China	Retrospective case-control	206	CD	Yes	X					X	X
*Fidan, 2017*	Turkey	Retrospective cohort	67	UC	No		X				X	X
*Fleshner, 2019*	USA	Prospective cohort	No data	UC	No					X		
*Gao, 2015*	China	Prospective case-control	110	CD	Yes	X	X				X	X
*Gao, 2020*	China	Retrospective cohort	54	CD	No				X		X	
*Gold, 2020*	USA	Retrospective cohort	107	UC, CD	No		X					
*Gur, 2018*	Turkey	Retrospective cohort	43	CD	No					X		
*Gur, 2020*	Turkey	Retrospective case-control	104	CD	Yes					X		
*Guthrie, 2013*	Scotland	Retrospective case-control	57	IBD	Yes	X						
*Hanafy, 2018*	Egypt	Prospective case-control	168	UC	Yes		X	X			X	X
*Hanai, 2004*	Japan	Prospective case-control	100	UC	Yes	X			X			
*Jardak, 2018*	Tunisia	Retrospective cohort	87	UC	No			X				
*Jeong, 2018*	South Korea	Retrospective case-control	174	IBD	Yes	X		X			X	X
*Jeong, 2021*	South Korea	Retrospective case-control	144	UC	Yes	X	X				X	
*Kang, 2017*	China	Retrospective cohort	108	CD	No			X		X	X	
*Khoury, 2019*	Israel	Retrospective case-control	436	CD	Yes					X	X	
*Messner, 2016*	Austria	Retrospective cohort	206	IBD	No							X
*Michalak, 2019*	Poland	Prospective case-control	112	UC	Yes	X						X
*Nassri, 2020*	USA	Retrospective cohort	No data	CD	No			X				
*Ndulue, 2019*	USA	Retrospective case-control	4739	IBD	Yes	X						
*Nishida, 2017*	Japan	Retrospective cohort	59	UC	No		X		X		X	
*Nishida, 2019*	Japan	Retrospective cohort	45	UC	No				X		X	
*Nishida, 2020*	Japan	Retrospective cohort	49	UC	No					X	X	
*Okba, 2019*	Egypt	Prospective case-control	80	UC	Yes	X	X	X			X	X
*Ovidiu, 2017*	Romania	Retrospective cohort	86	UC	No		X					
*Parisi, 2013*	Belgium	Retrospective cross-sectional	139	IBD	No					X		
*Posul, 2015*	Turkey	Prospective cohort	49	UC	No		X				X	
*Ryan, 2019*	USA	Uncontrolled, non-randomized trial	9	IBD	No				X			
*Stefanidis, 2015*	Greece	Retrospective cohort	35	IBD	No				X			
*Torun, 2012*	Turkey	Retrospective case-control	255	UC	Yes	X	X				X	X
*Wlodarczyk, 2015*	Poland	Retrospective case-control	45	CD	No				X		X	
*Xu, 2019*	China	Prospective cohort	214	UC, CD	No		X				X	
*Yamamoto-Furusho, 2020*	Japan	Retrospective cohort	158	UC	No		X	X			X	X
*Yarur, 2011*	USA	Retrospective cohort	68	IBD	No					X		
*Zhang, 2017*	China	Prospective case-control	34	UC, CD	Yes	X	X				X	
*Zhang, 2021*	China	Retrospective case-control	344	UC	Yes	X		X			X	
*Zhou, 2021*	China	Retrospective case-control	112	CD	Yes	X		X			X	

NLR: Neutrophil–lymphocyte ratio; UC: ulcerative colitis; CD: Crohn’s disease; IBD: inflammatory bowel disease; USA: United States of America. “X” denotes the aims identified in studies pertaining to NLR.

**Table 2:** Proposed NLR cutpoints by purpose and disease.

		Cutpoint Properties									Calculated Likelihood Ratios	
	Author, Year of Publication	Purpose	AUC	Cutpoint	SEN	SPE	PPV	NPV	OA	*p*-Value *	LR+	LR−
**Crohn’s Disease**	*Bou Jaoude, 2018*	Differentiate CD from non-CD	0.522	>1.98	0.684	0.431				>0.05	1.202	0.733
	*Chen, 2018*		0.828	2.85	0.692	0.762					2.908	0.404
	*Gao, 2015*		0.850	2.13	0.827	0.769					3.580	0.225
	*Feng, 2017*		0.740	2.72	0.683	0.759			0.701		2.834	0.418
	*Acarturk, 2015*	Differentiate active CD and remission (clinical)	0.830	3.20	0.810	0.590	0.930	0.740		<0.001	1.976	0.322
	*Ben Jeddi, 2019*		--	1.57							--	--
	*Chen, 2020*		0.764	3.32	0.659	0.759					2.734	0.449
	*Eraldemir, 2016*		0.703	2.58	0.696	0.760	0.727	0.731			2.900	0.400
	*Xu, 2019*		0.631	NR	NS	NS	NS	NS	NS		--	--
	*Zhang, 2017*		0.812	1.95	0.955	0.571	0.778	0.889	0.806		2.226	0.079
	*Zhang, 2017*	Differentiate severe and mild-to-moderate CD (clinical)	0.880	5.35	0.75	0.929	0.857	0.867	0.864	0.02	10.563	0.269
	*Khoury, 2019*	Part of a new clinical score to predict intra-abdominal masses	0.747	11.75 5.60	0.530 0.850	0.850 0.480					3.533 1.635	0.283 0.612
	*Crispino, 2021*	Predict endoscopic remission from biologic therapy at baseline	0.640	1.55	0.400	0.860	0.640	0.707		0.003	2.857	0.698
	*Ben Mustapha, 2015*	Predict sustained response to IFX therapy at baseline	--	<4.00	0.800	0.800				<0.05	4.000	0.250
	*Wlodarczyk, 2015*		0.850	4.07	0.800	0.870	0.860	0.810			6.154	0.230
	*Ben Mustapha, 2015*	Predict sustained response to IFX therapy at week 14	--	<3.50	0.720	0.700				<0.05	2.400	0.400
	*Wlodarczyk, 2015*		0.760	3.670	0.670	0.800	0.770	0.710			3.350	0.413
	*Gao, 2020*	Predict loss of response to IFX therapy at week 14	0.903	2.75	0.933	0.846				<0.00	6.058	0.079
	*Kang, 2017*	Predict postoperative complications	0.675	4.10	0.700	0.564					1.606	0.532
	*Cherfane, 2013*	Differentiate UC from non-UC	0.735	2.60	0.700	0.630					1.892	0.476
	*Dong, 2019*		0.731	4.70 *	0.613	0.857					4.287	0.452
**Ulcerative Colitis**	*Jeong, 2021*		0.774	2.26	0.542	0.906	0.578				5.766	0.506
	*Zhang, 2021*		0.858	2.66	0.750	0.826				<0.001	4.310	0.303
	*Acarturk, 2015*	Differentiate active UC and remission (clinical)	0.740	3.10	0.780	0.690	0.840	0.640		<0.001	2.516	0.319
	*Celikbilek, 2013*		--	2.47	0.539	0.632	0.667	0.500	0.578		1.465	0.729
	*Chen, 2020*		0.828	2.85	0.762	0.845					4.916	0.282
	*Demir, 2015*		0.640	2.39	0.486	0.775	0.680	0.604			2.160	0.663
	*Fidan, 2017*		0.722	2.20	0.620	0.700				<0.05	2.067	0.543
	*Hanafy, 2018*		0.810	2.35	0.740	0.860					5.286	0.302
	*Okba, 2019*		--	1.91	0.900	0.900					9.000	0.111
	*Posul, 2015*		0.650	2.30	0.612	0.667					1.838	0.582
	*Torun, 2012*		0.850	2.16	0.818	0.805	0.868	0.738			4.195	0.226
	*Xu, 2019*		0.625	NR	NS	NS	NS	NS	NS		--	--
	*Yamamoto-Furosho, 2020*		--	2.00	0.750	0.635					2.055	0.394
	*Zhang, 2017*		0.726	3.29	0.474	0.939	0.900	0.583	0.676		7.770	0.560
	*Jeong, 2021*	Differentiate severe and mild-to-moderate UC (clinical)	0.714	3.44	0.636	0.811					3.365	0.449
	*Zhang, 2017*		0.560	3.92	0.375	1.000	1.000	0.231	0.474	0.517		0.625
	*Akpinar, 2018*	Differentiate active UC and remission (endoscopic)	0.718	2.42	0.760	0.702				0.003	2.550	0.342
	*Zhou, 2021*		0.680	4.45	0.839	0.469	0.522	0.809	0.62	< 0.001	1.580	0.343
	*Yamamoto-Furosho, 2020*		--	2.09	0.639	0.588					1.551	0.614
	*Cherfane, 2013*	Differentiate active UC from *C. difficile* infection	0.693	3.10	0.700	0.650					2.000	0.462
	*El-Sadek, 2021*	Predict UC flare during pregnancy	0.915	2.85	0.900	0.882				0.001	--	--
	*Nishida, 2021*	Predict development of pouchitis after ileal pouch-anal anastomosis	0.680	2.15	0.722	0.677					--	--
	*Bertani, 2019*	Predict clinical remission with anti-TNF medications at baseline	0.889	2.33	0.900	0.650					2.571	0.154
	*Bertani, 2019*	Predict mucosal healing with anti-TNF medications at baseline	0.853	2.33	0.800	0.6700					2.424	0.299
	*Bertani, 2020*		--	2.06	0.600	0.792					2.885	0.505
	*Nishida, 2017*	Predict response to IFX therapy at baseline	0.702	4.49	0.786	0.783					3.622	0.273
	*Nishida, 2019*	Predict risk of relapse with tacrolimus therapy at baseline	--	5.84	0.625	0.667					1.877	0.562
**IBD**	*Jeong, 2018*	Differentiate IBD from non-IBD	0.802	1.80	0.707	0.733					2.648	0.400
	*Chalmers, 2017*	Differentiate PIBD from non-IBD	0.810	2.37	0.67	0.85					4.467	0.388

NLR: Neutrophil–lymphocyte ratio; CD: Crohn’s disease; IFX: infliximab; UC: ulcerative colitis; TNF: tumor necrosis factor (alpha); IBD: inflammatory bowel disease; PIBD: pediatric inflammatory bowel disease; NR: not reported due to lack of statistical significance; NS: non-significant; AUC: area under the curve; SEN: sensitivity; SPE: specificity; PPV: positive predictive value; NPV: negative predictive value; OA: overall accuracy; LR+: likelihood ratio positive; LR−: likelihood ratio negative. * *p*-values for discrimination between groups using the cutpoint for NLR using receiver operative curve analysis; ** Note: the original manuscript reported an NLR cutpoint value of 0.470 which we assume to be a typographical error related to decimal placement. We have unsuccessfully reached out to the authors to confirm.

**Table 3:** Association of NLR with other IBD biomarkers.

Study Population		NLR Associations & Correlations								
	Author, Year of Publication	CRP	ESR	WBC	PLR	Fibrinogen	Fecal Calprotectin	Fecal Lactoferrin	Malondialdehyde	Nitric Oxide
**Crohn’s Disease**	*Acarturk, 2015*	* r_s_ = –0.61, *p* = 0.793 ** r_s_ = −0.022, *p* = 0.924	* r_s_ = 0.242, *p* = 0.291 ** r_s_ = −0.042, *p* = 0.856	* r_s_ = 0.242, *p* ≤ 0.001 ** r_s_ = −0.135, *p* = 0.561						
	*Eraldemir, 2016*	* B = −0.044, 95% CI −0.205–0.116, *p* = 0.573	* B = 0.174, 95% CI −0.044–0.393, *p* = 0.112						* B = 0.422, 95% CI 0.0480.796, *p* = 0.029	
	*Feng, 2017*	r_s_ = 0.39, *p* < 0.01	r_s_ = 0.43, *p* < 0.01							
	*Gao, 2015*	r_s_ = 0.327, *p* < 0.001	r_s_ = 0.137, *p* = 0.082	r_s_ = 0.493, *p* < 0.001						
**Ulcerative Colitis**	*Acarturk, 2015*	* r_s_ = 0.116, *p* = 0.463 ** r_s_ = −0.198, *p* = 0.208	* r_s_ = 0.051, *p* = 0.750 ** r_s_ = 0.200, *p* = 0.203	* r_s_ = 0.260, *p* = 0.096 ** r_s_ = 0.266, *p* = 0.089						
	*Bertani, 2019*				NS		NS			
	*Bertani, 2020*						r_s_ = 0.11 (baseline), *p* > 0.05 r_s_ = 0.21 (week 8), *p* > 0.05			
	*Demir, 2015*	r_s_ = 0.185, *p* = 0.059 * r_s_ = 0.141, *p* = 0.246 ** r_s_ = 0.020, *p* = 0.911	r_s_ = 0.170, *p* = 0.043 * r_s_ = 0.121, *p* = 0.319 ** r_s_ = 0.088, *p* = 0.468	r_s_ = 0.282, *p* = 0.001 * r_s_ = 0.360, *p* = 0.002 ** r_s_ = 0.097, *p* = 0.420						
	*Dong, 2019*	* *p* < 0.05	* *p* < 0.05							
	*El-Sadek, 2019*	r_s_ = 0.418, *p* = 0.03	r_s_ = 0.522, *p* = 0.005							
	*Eraldemir, 2014*								NS	r^2^ = 0.593, *p* < 0.001
	*Fidan, 2017*			* r_s_ = 0.370, *p* < 0.05	* r_s_ = 0.944, *p* < 0.05					
	*Hanafy, 2018*							*p* < 0.001		
	*Michalak, 2019*				*p* < 0.001					
	*Okba, 2019*	r_s_ = 0.789, *p* < 0.001 * r_s_ = 0.490, *p* = 0.028 ** r_s_ = 0.146, *p* = 0.538	r_s_ = 0.556, *p* < 0.001 * r_s_ = 0.597, *p* = 0.005 ** r_s_ = −0.139, *p* = 0.558	r_s_ = 0.324, *p* = 0.012 * r_s_ = 0.184, *p* = 0.437 ** r_s_ = 0.088, *p* = 0.712						
	*Torun, 2012*	r_s_ = 0.102, *p* = 0.153	r_s_ = 0.217, *p* = 0.002	r_s_ = 0.416, *p* < 0.001		r_s_ = 0.095, *p* = 0.187				
	*Yamamoto-Furosho, 2020*						r_s_ = 0.347, *p* < 0.001			
**IBD**	*Jeong, 2018*	r^2^ = 0.348, *p* = 0.008								
	*Messner, 2016*						r^2^ = 0.210, *p* ≤ 0.05			

NLR: Neutrophil–lymphocyte ratio; NS: non-significant (correlation statistics and *p*-values not reported); r_s_: Spearman’s r coefficient; r^2^: Pearson’s r-squared coefficient; B: regression coefficient; CI: confidence interval; CRP: c-reactive protein; ESR: erythrocyte sedimentation rate; PLR: platelet-lymphocyte ratio; WBC: total white blood cell count. * Value for active disease, specifically; ** Value for inactive disease, specifically.

## Data Availability

No new data were created or analyzed in this review. Data sharing is not applicable to this article.

## Supplementary Materials

The following are available online at https://www.mdpi.com/article/10.3390/jcm10184219/s1, Preferred Reporting Items for Systematic reviews and Meta-Analyses extension for Scoping Reviews (PRISMA-ScR) Checklist.

## Appendix A

(Inflammatory Bowel Diseases[mh] OR inflammatory bowel disease*[tiab] OR IBD[tiab] OR crohn*[tiab] OR “ulcerative colitis”[tiab]) AND (((Neutrophils[mh] OR neutrophil*[tiab]) AND (Lymphocytes[mh] OR Lymphocyte Count[mh] OR lymphocyte*[tiab])) OR ((“neutrophil to lymphocyte”[tiab] OR “neutrophil lymphocyte”[tiab]) AND (ratio[tiab] OR rate[tiab] OR count[tiab]))).
